# Supplementary material for: Purification and Biochemical Characterization of the DNA Binding Domain of the Nitrogenase Transcriptional Activator NifA from Gluconacetobacter diazotrophicus
Source: Protein J. 2023 Oct 3;42(6):802–10. doi: 10.1007/s10930-023-10158-w (PMC10590331; doi:10.1007/s10930-023-10158-w)
Supplement: Supplementary file 1 — Supplementary material 1 (DOCX 663.4 kb) [file 10930_2023_10158_MOESM1_ESM.docx]

**Supporting information for:**

**Purification and biochemical characterization of the DNA binding domain of the nitrogenase transcriptional activator NifA from *Gluconacetobacter diazotrophicus***

**Heidi Standke^‡^, Lois Kim^‡^, Cedric P. Owens^‡^***

**^‡^**Schmid College of Science and Technology

Chapman University,

One University Drive

Orange, CA 92866

USA

***Corresponding Author**

Cedric Owens

Chapman University

Schmid College of Science and Technology

Keck Center for Science and Engineering 226

One University Drive, Orange, CA 92866

cpowens@chapman.edu

Phone: 001-714-997-6922

**Figure S1.** Representative standard curve for Ellman’s assay using L-Cys as a standard.


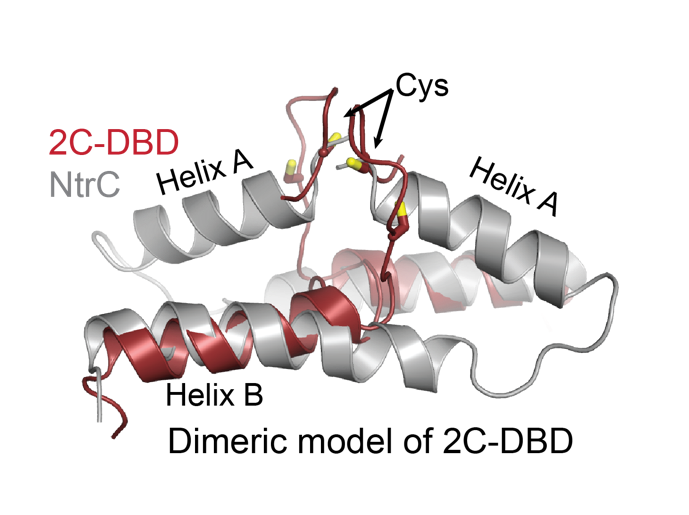


**Figure S2.** Model of dimeric 2C-DBD aligned with dimeric NtrC (pdb id: 1ntc). Possible orientations of the flexible IDL region in 2C-DBD were modeled using the MoMA loop sampler, revealing IDL orientations that place Cys resides of different protomers in proximity to each other. The 2C-DBD dimer was created by aligning two copies of 2C-DBD with the two chains in the NtrC dimer. For clarity, only the IDL, Helix A (present only in NtrC), and Helix B are depicted.


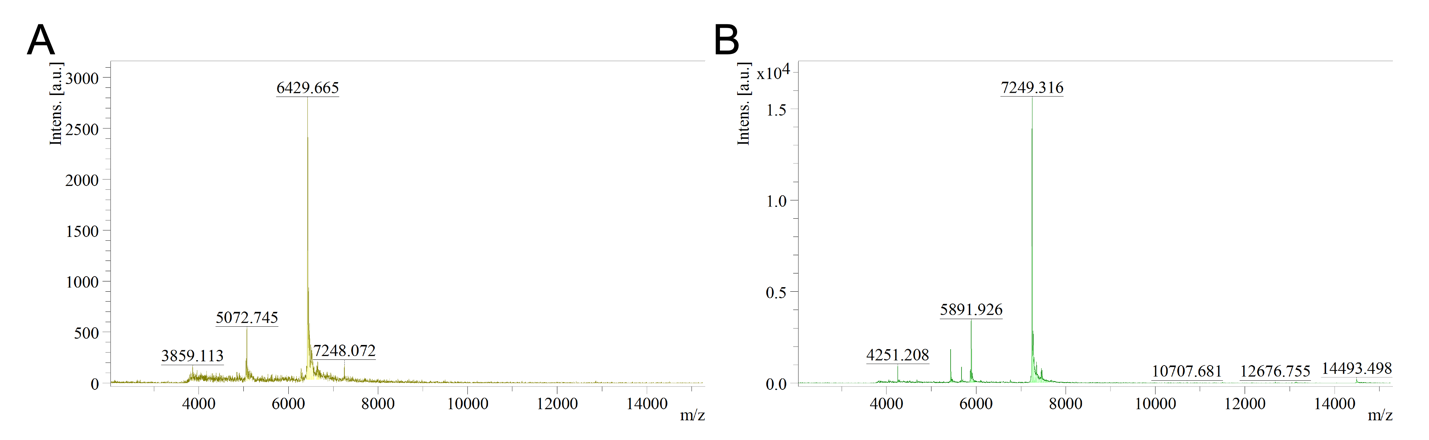


**Figure S3.** MALDI-TOF of (A) NC-DBD and (B) 2C-DBD after His-tag cleavage. The expected masses are 6428.4 g/mol and 7248.3 g/mol for [NC-DBD + H]^+^ and [2C-DBD + H]^+^, respectively. The 1 m/z mass difference between predicted and measured value is within the error of the instrument.


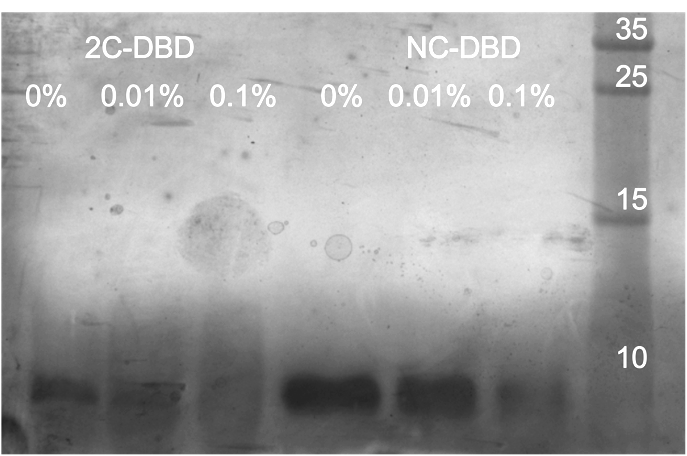


**Figure S4.** Glutaraldehyde crosslinking of 2C-DBD and NC-DBD indicating that no dimer or higher order oligomers are formed in presence of the nonspecific crosslinker glutaraldehyde. Glutaraldehyde concentrations are listed above each lane. The gel was silver stained to detect potential small amounts of dimer which would appear at 13 kDa for NC-DBD and 14.5 kDa for 2C-DBD. The gel is representative of three independent replicates. The rightmost lane represents the molecular weight marker with standards in kDa.


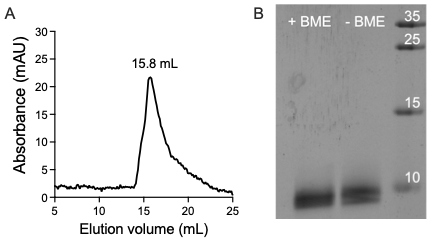


**Figure S5.** (A) Analytical gel filtration chromatograms of 2C-DBD run in presence of 5 mM TCEP. Results are identical when 10 mM DTT was used as a reducing agent instead of TCEP. (B) SDS-PAGE of 2C-DBD that was reduced with 5% BME and 2C-DBD that was not reduced. The migration distance is the same for both samples, indicating that the Cys residues do not form an intermolecular disulfide. The rightmost lane is the molecular weight marker with standards in kDa.

**Figure S6.** (A) Controls indicating that the DNA binding domain does not bind nonspecifically to the fluorescent probe since excess unlabeled DNA will outcompete the fluorescently labeled DNA, causing anisotropy to decrease. (B) Control indicating that the DNA binding domain does not bind nonspecifically to DNA. Adding a scrambled nifH-UAS duplex (5’AGTCGTCAAGGCTCGTCCTCGATGAGTC) to 2C-DBD does not cause a concentration-dependent change in fluorescence, suggesting that scrambled DNA does not compete with labeled nifH-UAS for 2C-DBD binding. The protein concentration in (A) and (B) was 50 µM and the labeled DNA concentration was 900 nM. (C) Control experiments demonstrating that the nifH-UAS probe (900 nM) does not bind nonspecifically to BSA.
